# Is there an association between metabolic syndrome and drug resistance in patients with epilepsy?

**DOI:** 10.1097/MD.0000000000050264

**Published:** 2026-08-21

**Authors:** Afshan Davari, Amir Reza Bahadori, Azadeh Imeni Kashan, Mehrdad Sheikhvatan, Sara Ranji, Sajad Shafiee, Abbas Tafakhori

**Affiliations:** a Medical Colleges, Tehran University of Medical Sciences, Tehran, Iran; b Iranian Center of Neurological Research, Neuroscience Institute, Tehran University of Medical Sciences, Tehran, Iran; c School of Medicine, Shiraz University of Medical Sciences, Shiraz, Iran; d School of Medicine, Iran University of Medical Sciences, Tehran, Iran; e Medical Biology and Genetics Department, Okan University, Istanbul, Turkey; f Mazandaran University of Medical Sciences, Sari, Iran.

**Keywords:** drug-resistant epilepsy, epilepsy, metabolic syndrome, prevalence, risk factors

## Abstract

Epilepsy is one of the most prevalent neurological disorders, affecting approximately 1% of the population, with one-third of patients developing drug-resistant epilepsy (DRE). In addition, metabolic syndrome (MetS), through inflammation and oxidative stress mechanisms, may influence epilepsy outcomes. The main objective of the current study is to determine the prevalence of MetS among patients with epilepsy (PWE) and evaluate its association with DRE. This cross-sectional study was conducted between March and October 2024 at a regional hospital in Tehran, involving 180 PWE aged 18 to 70 years. Participants were classified into well-controlled epilepsy and DRE groups based on the International League Against Epilepsy criteria. MetS was also diagnosed using the International Diabetes Federation guidelines. Data were analyzed using SPSS software, which illustrated that MetS was present in 33.8% of participants, with a significantly higher prevalence in well-controlled epilepsy patients (47.7%) compared with those with DRE (20%, *P* < .001), and it played a role in predicting response to treatment in PWE. Furthermore, body mass index, waist circumference, and blood pressure were significantly higher in the well-controlled group (*P* = .012 and *P* = .007, respectively). MetS was more prevalent in well-controlled epilepsy patients and was associated with a decreased risk of DRE. However, further research is needed to explore the relationship between MetS and DRE.

## 1. Introduction

Epilepsy is one of the most common brain diseases that, regardless of gender, age, and race, affects 5 to 10 individuals per 1000 yearly.^[1]^ Despite the availability of diverse therapeutic regimens for epilepsy management, one-third of patients exhibit inadequate responses to these interventions, failing to achieve sustained seizure remission.^[2]^ In such cases, the International League Against Epilepsy (ILAE) defined drug-resistant epilepsy (DRE) as the failure of adequate trials of 2 tolerated, appropriately chosen, and appropriately used antiseizure medications (ASMs), whether as monotherapies or in combination, to achieve sustained seizure freedom.^[3]^ DRE is associated with a heavy healthcare-related economic burden.^[4,5]^ Therefore, the identification of risk factors that play a role in DRE assumes importance.^[3,5]^

Metabolic syndrome (MetS), according to the International Diabetes Federation (IDF), is defined by the presence of 3 or more of the following parameters: insulin resistance, low degree of high-density lipoprotein cholesterol (HDL-C), elevated triglycerides (TG), central obesity, and hypertension.^[6]^ MetS should be distinguished from the metabolic etiology of epilepsy as defined by the ILAE classification, where seizures are a core symptom of a specific metabolic disorder.^[7]^ MetS contributes to the development of a proinflammatory and oxidative stress state within the body.^[8]^ On the other hand, Hayatdavoudi et al^[9]^ demonstrated that inflammation had a significant impact on the pathophysiology of epilepsy by disrupting the blood–brain barrier, which maintains a stable environment for cells of the central nervous system, and stimulating astrocytes involved in regulating ion channels and neuronal depolarization. It appears that these 2 conditions are linked through inflammation. As seizure control represents a significant challenge for epilepsy patients,^[10]^ it is essential to ascertain the determinants of seizure control.

Furthermore, several studies indicated that a significant association was observed between individuals with epilepsy who are obese and may have a higher risk of developing DRE.^[11,12]^ On the other hand, repeated seizures create an extraordinary metabolic burden,^[13]^ and this increase in energy expenditure may lead to weight loss.^[14]^ So, further research is needed to investigate this correlation between seizure and MetS. Therefore, the main aim of the present cross-sectional study is to estimate the prevalence of MetS among individuals who suffer from epilepsy and examine the probable association between MetS and response to ASM among them.

## 2. Methods and materials

### 2.1. Study design and participants

The present cross-sectional study included adult patients (18–70 years) with a confirmed diagnosis of epilepsy according to the criteria of the ILA. Participants were recruited from both outpatient and inpatient neurology services at a regional hospital in Tehran between March 2024 and October 2024. All patients were under regular neurologic follow-up for at least 1 year and were receiving ASMs, with adherence to treatment confirmed by neurologist assessment. Both focal and generalized epilepsies were included.

DRE was defined according to ILAE criteria as the failure of adequate trials of 2 tolerated, appropriately chosen, and appropriately used ASMs (whether as monotherapy or in combination) to achieve sustained seizure freedom.^[3]^ Based on these criteria and neurologist assessment, participants were classified into either the DRE or well-controlled epilepsy group.

Sample size was estimated based on the reported prevalence of obesity in epilepsy,^[15]^ yielding an effect size (Cohen’s h ≈ 0.55). With a significance level of α = 0.05 and 80% statistical power, a minimum of 140 participants (70 per group) was required to detect differences in MetS prevalence between groups. A total of 180 patients (80 females and 100 males) were ultimately enrolled, exceeding the required sample size and ensuring adequate statistical power.

Exclusion criteria were postmenopausal or pregnant women (due to metabolic alterations); individuals with endocrine disorders other than diabetes mellitus; active malignancy; current use of systemic corticosteroids; use of medications affecting body weight or metabolism; and documented nonadherence to ASM therapy.

### 2.2. Data gathering

Demographic and seizure-related parameters of included subjects, such as age, sex, education level, past medical history, past drug history of ASM, seizure attack-free days, duration of each seizure attack, seizure attack frequency in the past month, and the Seizure Severity Questionnaire, were gathered.

In addition, anthropometric measurements including weight (with a precision of 0.1 kg) in light clothing using a Seca scale, height (with a precision of 0.5 cm) using a wall-mounted stadiometer, and waist circumference (WC to the nearest 0.5 cm) using a measuring tape at the level of midway between the lower costal margin and the iliac crest were obtained by a trained assessor. Body mass index (BMI) was calculated by dividing weight in kilograms by height in meters squared. Blood pressure (BP) was measured using a sphygmomanometer. Serum triglyceride (TG), HDL-C, total cholesterol (T-chol), low-density lipoprotein cholesterol (LDL-C), and fasting blood sugar (FBS) levels were measured from blood samples collected from participants after a 12 to 14 hour fasting. Or FBS were collected from participants after a 12 to 14 hour for the measurement of serum TG, HDL-C, LDL-C, T-chol, and FBS levels fast.

### 2.3. Metabolic syndrome: definition

MetS was diagnosed according to the harmonized definition proposed in the 2009 Joint Interim Statement developed in collaboration with the International Diabetes Federation.^[6]^ Based on this definition, participants with at least 3 or more of the following abnormalities were classified as having MetS: FBS ≥100 mg/dL or previously diagnosed diabetes; HDL-C <40 mg/dL in men and <50 mg/dL in women, or drug treatment for low HDL-C; TG ≥150 mg/dL or drug treatment for elevated TG; BP ≥130/85 mm Hg or antihypertensive treatment; and abdominal obesity defined as waist circumference ≥94 cm for men and ≥80 cm for women.

### 2.4. Statistical analysis

All data were entered into Microsoft Excel and then analyzed using SPSS Statistics for Windows (Version 23.0; IBM Corp.). Qualitative variables were presented as frequency and percent, and quantitative variables as mean ± standard deviation. Differences between groups were assessed using the chi-square test for categorical variables. Continuous variables were first tested for normality using the Shapiro–Wilk test; normally distributed variables were analyzed with the independent-samples *t* test, and non-normally distributed variables with the Mann–Whitney *U* test. Clinically relevant variables, including age, gender, onset, and MetS, were considered for inclusion in the multivariable logistic regression model. Variables causing multicollinearity or unstable estimates were excluded to maintain model stability. Logistic regression analysis was then performed to identify factors predicting the development of DRE. Results were considered statistically significant at a two-tailed *P* value <.05.

### 2.5. Ethics approval

It should be mentioned that this current study has been registered with the registration number 70826 in the office of the Institute of Board Review of *Tehran University of Medical Sciences, Tehran, Iran,* and approved by the Ethics Committee of Research under the number: IR.TUMS.IKHC.REC.1403.083. In addition, informed consent was obtained from each participant before their involvement in the study.

## 3. Results

The mean age of the total 180 participants was 34.58 ± 11.50 years, and 44.4% were women. The individuals’ average age at onset of epilepsy was 18.09 ± 12.75 years, with a median duration of epilepsy of 15.5 years. According to ILAE criteria, and a neurologist’s assessment, 90 patients suffer from DRE, and the other half of participants were patients with well-controlled epilepsy. Statistically significant differences were observed in age (*P* = .008) and gender (*P* = .036) between the groups. Among the well-controlled epilepsy groups, 15 patients were on monotherapy with valproic acid, 6 were on carbamazepine, 33 were on levetiracetam, and the remaining individuals were on polytherapy with 2 or more ASMs.

Based on the IDF definition of MetS, 28 (15.5%) of the population studied had FBS ≥100 (6 of well-controlled epilepsy and 22 of DRE), 44 (24.4%) had TG ≥150 (26 of well-controlled epilepsy and 18 of DRE), 45 (25%) had BP ≥130/85 (36 of well-controlled epilepsy and 9 of DRE), 42 (52.5%) of women had HDL-C <50, and 48 (48%) of men had HDL-C <40 (51 of well-controlled epilepsy and 39 of DRE). In addition, 49 (61.2%) of women had a waist circumference (WC) ≥80 and 57 (57%) of men had a WC ≥94 (64 of well-controlled epilepsy and 42 of DRE). Taken together, 61 (33.8%) of patients with epilepsy (PWE) reported having MetS. The mean BMI of the participants was 23.86 ± 4.25 kg/m^2^. Among the PWE, 45 (25%) were classified as overweight (BMI 25–29.9 kg/m^2^) and 19 (10.5%) as obese (30 ≤ BMI < 35 kg/m^2^).

Mean FBS was 88.68 ± 14.46 mg/dL, with a median (interquartile range) of total cholesterol 169.5 (143.25–188.75) mg/dL, TG 106 (74.25–152.5) mg/dL, LDL 97 (74–112) mg/dL, and HDL 44 (36–54) mg/dL. A comparison between the well-controlled epilepsy and DRE groups is shown in Table 1. The prevalence of MetS was significantly higher in patients with well-controlled epilepsy compared with the DRE group (*P* < .001). Also, a significant difference was observed between the well-controlled epilepsy and DRE groups in terms of weight, BMI, WC, and BP, with higher values found in the well-controlled epilepsy group (*P* = .008, *P* = .011, *P* < .001, and *P* < .001, respectively).

**Table 1:** Comparative analysis of study variables between well-controlled epilepsy and drug-resistant epilepsy.

Variables	All participants (n = 180)	Well-controlled epilepsy (n = 90)	Drug-resistant epilepsy (n = 90)	*P*
Sex, N (%)				
Male	100 (55.6)	58 (64.4)	42 (46.7)	.036*^,^†
Female	80 (44.4)	32 (35.5)	48 (53.3)	
Education, N (%)				
Illiterate	15 (8.3)	11 (12.2)	4 (4.4)	.084†
Undergraduate	78 (43.3)	36 (40)	42 (46.7)	
Diploma	60 (33.3)	33 (36.7)	27 (30)	
Academic degree	27 (15)	8 (8.8)	19 (21.1)	
Age (yr)	34.58 ± 11.50	36.83 ± 12.80	32.33 ± 9.59	.008*^,^‡
Height (cm)	167.65 ± 7.92	168.43 ± 7.55	166.86 ± 8.24	.185‡
Weight (kg)	67.43 ± 14.54	70.33 ± 15.64	64.63 ± 12.80	.008*^,^‡
BMI (kg/m^2^)	23.86 ± 4.25	24.66 ± 4.76	23.06 ± 3.52	.011*^,^‡
Overweight, N (%)	45 (25)	30 (33.3)	15 (16.7)	.015*^,^†
Obese, N (%)	19 (10.5)	12 (13.3)	7 (7.7)	.213†
WC (cm)	87.15 ± 11.47	90.83 ± 11.59	83.46 ± 10.13	< .001*^,^‡
SBP (mm Hg)	116.46 ± 12.97	119.83 ± 15.76	113.10 ± 8.20	< .001*^,^‡
DBP (mm Hg)	77.1 ± 8.97	80.13 ± 8.68	74.06 ± 8.23	< .001*^,^‡
BP ≥ 130/85 (mm Hg), N (%)	46 (25.5)	37 (41.1)	9 (10)	< .001*^,^†
FBS (mg/dL)	88.68 ± 14.46	89.03 ± 12.04	88.33 ± 16.59	.747‡
TG (mg/dL)	106 (74.25–152.5)	88.5 (72–156)	109 (82–143)	.116§
HDL-C (mg/dL)	44 (36–54)	44 (36–48)	44 (36–58)	.138§
LDL-C (mg/dL)	97 (74–112)	93.5 (77–109)	104.5 (74–115)	.091§
T-chol (mg/dL)	169.5 (143.25–188.75)	159 (136–180)	172.5 (150–198)	.013*^,^§
Metabolic syndrome, N (%)	61 (33.8)	43 (47.7)	18 (20)	< .001*^,^†
Onset (age of diagnosis)	16.5 (9–25)	21 (16–31)	9 (3–19)	< .001*^,^§
Duration of disease (yr)	15.5 (6.25–24.75)	8.5 (3–20)	21 (14–27)	< .001*^,^§
Duration of attack (min)	1 (1–2)	1 (1–2)	2 (1–2.5)	.009*^,^§
Frequency/mo	0.25 (0–7.75)	0	7.5 (3–13)	< .001*^,^§
Free seizure day	90 (4–496.25)	452 (330–730)	4 (1–9)	< .001*^,^§
SSQ total score	43.49 ± 18.50 (n = 153)	37.23 ± 17.72 (n = 63)	47.86 ± 17.64 (n = 90)	**< .001**‡
Drug, N (%)				
VPA	78 (44.1)	36 (40.0)	42 (48.3)	.292†
CBZ	69 (38.3)	18 (20.0)	51 (56.7)	< .001*^,^†
LCM	45 (25.0)	15 (33.3)	30 (66.7)	.015*^,^†
LEV	120 (66.7)	54 (45.0)	66 (55.0)	.082†
TPM	13 (7.2)	0	13 (14.4)	< .001*^,^†
CLB	12 (6.7)	6 (6.7)	6 (6.7)	1.000†
LTG	43 (23.8)	6 (6.7)	37 (41.1)	< .001*^,^†
PHB	9 (5.0)	0	9 (10.0)	.003*^,^†

Values are given as mean ± standard deviation except for TG, T-chol, HDL-C, LDL-C, onset, duration, frequency/month, free seizure day given as median (Q1–Q3).

BMI = body mass index, DBP = diastolic blood pressure, FBG = fasting blood glucose, HDL-C = high-density lipoprotein cholesterol, LDL-C = low-density lipoprotein cholesterol, N = number, SBP = systolic blood pressure, SSQ = Seizure Severity Questionnaire, T-chol = total cholesterol, TG = triglyceride, WC = waist circumference.

† Pearson chi-square test.

‡ Impaired sample *t*-test.

§ Mann–Whitney *U* test.

* *P* < .05 was considered statistically significant.

Compared with the well-controlled group, individuals with DRE had an earlier age at seizure onset (*P* < .001) and reported higher seizure severity based on the Seizure Severity Questionnaire (*P* < .001). Also, the duration of attacks was longer in the DRE group compared with the other group (*P* < .001).

In addition, owing to multivariate analysis, it is common for the significance of predictive variables to change. Therefore, many studies include relevant and potentially predictive variables in their analysis.^[15]^ Using this criterion, logistic regression analysis revealed that the presence of MetS was associated with a decreased risk of DRE (*P* = .001, *R* = 0.266) (Table 2).

**Table 2:** The multivariable logistic regression model in determining the role of metabolic syndrome in predicting drug-resistant epilepsy.

Factor	Beta	*P*	OR	95% CI	
				Lower	Upper
Gender	0.198	.617	1.219	0.561	2.650
Age	0.042	**.033** *	1.043	1.004	1.084
Onset	−0.128	**< .001** *	0.880	0.842	0.920
Metabolic syndrome	−1.132	**.001** *	0.266	0.123	0.574

*Hosmer–Lemeshow goodness of fit: Chi-square = 12.24, P = .141.*

* *P* < .05 was considered statistically significant.

## 4. Discussion

The current cross-sectional study was carried out on the prevalence of MetS among PWE. Furthermore, we also evaluated how this prevalence differed between 2 subgroups of well-controlled epilepsy and DRE.

One-third of our study population was categorized as MetS, and a significant difference was shown in MetS prevalence between well-controlled epilepsy and DRE groups, with higher rates in the well-controlled group. According to logistic regression analysis, the presence of MetS, which was adjusted for sex, age, and onset of epilepsy, was associated with better control of epilepsy among individuals. However, this should be interpreted as a correlation rather than evidence of a causal relationship.

Our findings are similar to the 32.6 percent of MetS prevalence in Söylemez et al research.^[16]^ Most studies examining the prevalence of MetS in seizure patients have not reported prevalence based on the response to ASMs, and the study populations were a mix of well-controlled and DRE patients.^[17–19]^ While Vooturi’s study, which exclusively examined patients with well-controlled epilepsy, reported a 52.6% prevalence of MetS, the highest observed.^[20]^ Our findings corroborate these results, revealing a significantly higher prevalence of 47.7% in our well-controlled PWE compared with the 20% observed in those with refractory epilepsy.

Some studies have suggested a link between MetS and epilepsy.^[17,18,20]^ Potential factors include changes in inflammatory markers, adiponectin levels, and energy metabolism.^[21,22]^

In epilepsy, disruptions in glucose and lipid metabolism can trigger inflammatory pathways, which in turn may increase neuronal excitability and contribute to disease progression.^[23]^ In line with this, clinical research has shown that adults with epilepsy—even those newly diagnosed and not yet on antiepileptic medication—tend to have higher BMI and obesity rates compared with healthy individuals. These metabolic changes are influenced by factors such as age, gender, epilepsy etiology, and inflammatory markers.^[24]^ Altogether, these findings provide a plausible biological explanation for the association we observed between MetS and treatment response in our study.

The association of MetS and response to ASMs has rarely been investigated. Although the prevalence of MetS varied between the two groups, a significant association was found between MetS and DRE, it important to note that this observational study should be viewed as hypothesis-generating, and further longitudinal research is required to confirm causal relationships. This discrepancy in prevalence may be attributed to the fact that certain ASMs—for instance, carbamazepine, topiramate, and lamotrigine, compared with others that can contribute to MetS,^[25]^ may not influence or may even reduce values of factors contributing to MetS and anthropometric indices such as BP, waist circumference, BMI, and insulin resistance.^[26–30]^ Potential mechanisms include reducing appetite and subsequent caloric intake, decreasing leptin levels while increasing adiponectin levels, enhancing insulin sensitivity, and influencing blood electrolyte concentrations.^[31–34]^ In our study population, those with DRE exhibited significantly higher consumption of these medications compared with the well-controlled group. The underlying mechanisms linking MetS to epilepsy and treatment response are complex and require further investigation.

Although our study did not stratify patients based on epilepsy type (focal vs generalized) or etiology, this represents an important area for future investigation. Detailed classification would require consistent access to neuroimaging and electroencephalography data, which were not uniformly available in our cohort. Future studies should aim to incorporate these clinical variables to better understand how epilepsy subtype and underlying etiology may influence the prevalence and impact of MetS.

The clinical implications of our findings are significant, as higher WC and high BP have a higher risk of developing cardiovascular diseases such as coronary heart disease or stroke.^[35–37]^ Therefore, PWE, especially those with well-controlled seizures, should be closely monitored for the development of MetS, particularly the 2 components of MetS definition criteria, WC and BP, which were significantly higher in participants with well-controlled epilepsy. Early diagnosis and management of MetS can help reduce the risk of cardiovascular complications and improve overall health outcomes.^[38]^

While our study is the first to comprehensively examine the prevalence of MetS and its relationship to treatment response in PWE, it is not without limitations. The cross-sectional design hindered our ability to analyze how different ASMs affect MetS among participants with epilepsy. Nonetheless, future studies should employ a prospective cohort design with a larger sample size to further investigate this relationship. Moreover, exploring other relevant factors, such as physical activity, and including a healthy control group would enhance the robustness of the findings. In addition, certain clinically relevant variables, such as detailed ASM subtypes and seizure frequency, were not included in the multivariable analysis due to limited subgroup sizes and concerns regarding model instability and overadjustment. Future studies should also consider stratifying ASMs based on their enzyme-inducing properties to better understand their potential impact on lipid metabolism and the development of MetS in PWE. In addition, our study included both well-controlled and drug-resistant patients. However, all participants were recruited from a single tertiary referral center, which may overrepresent patients with more complex clinical profiles. Therefore, caution is needed when generalizing these findings to community-based or primary care populations.

## 5. Conclusion

In conclusion, our study results demonstrated that nearly one-third of individuals with epilepsy have MetS according to IDF 2009 criteria. The prevalence of MetS was significantly higher in well-controlled epilepsy patients (47.7%) compared with the DRE group (20%), and a role can be expected for MetS in predicting response to treatment in PWE. Further cohort-designed studies are needed to investigate the interaction between MetS and the development of DRE.

## Acknowledgments

We are sincerely thankful to all the individuals who participated in this study.

## Author contributions

**Conceptualization:** Afshan Davari, Mehrdad Sheikhvatan, Abbas Tafakhori.

**Data curation:** Afshan Davari.

**Formal analysis:** Afshan Davari, Mehrdad Sheikhvatan.

**Project administration:** Afshan Davari, Amir Reza Bahadori, Azadeh Imeni Kashan, Sajad Shafiee.

**Writing – original draft:** Afshan Davari.

**Writing – review & editing:** Amir Reza Bahadori, Abbas Tafakhori.

**Supervision:** Sara Ranji, Abbas Tafakhori.

## References

[1] Fiest KM Sauro KM Wiebe S. Prevalence and incidence of epilepsy: a systematic review and meta-analysis of international studies. Neurology. 2017;88:296–303. 27986877 10.1212/WNL.0000000000003509 PMC5272794

[2] Sultana B Panzini M-A Veilleux Carpentier A. Incidence and prevalence of drug-resistant epilepsy: a systematic review and meta-analysis. Neurology. 2021;96:805–17. 33722992 10.1212/WNL.0000000000011839

[3] Kwan P Arzimanoglou A Berg AT. Definition of drug resistant epilepsy: consensus proposal by the ad hoc Task Force of the ILAE Commission on Therapeutic Strategies. Epilepsia. 2010;51:1069–77. 19889013 10.1111/j.1528-1167.2009.02397.x

[4] Bahadori AR Javadnia P Mohseni G. Prevalence of drug-resistant epilepsy in low and middle income countries: a systematic review and meta-analysis. Epilepsy Behav. 2026;183:111135. 42218854 10.1016/j.yebeh.2026.111135

[5] Janson MT Bainbridge JL. Continuing Burden of Refractory Epilepsy. SAGE Publications; 2021:406–8. 10.1177/1060028020948056 32795085

[6] Alberti KG Eckel RH Grundy SM. Harmonizing the metabolic syndrome: a joint interim statement of the International Diabetes Federation Task Force on Epidemiology and Prevention; National Heart, Lung, and Blood Institute; American Heart Association; World Heart Federation; International Atherosclerosis Society; and International Association for the Study of Obesity. Circulation. 2009;120:1640–5. 19805654 10.1161/CIRCULATIONAHA.109.192644

[7] Scheffer IE Berkovic S Capovilla G. ILAE classification of the epilepsies: position paper of the ILAE Commission for Classification and Terminology. Epilepsia. 2017;58:512–21. 28276062 10.1111/epi.13709 PMC5386840

[8] Reddy P Lent-Schochet D Ramakrishnan N McLaughlin M Jialal I. Metabolic syndrome is an inflammatory disorder: a conspiracy between adipose tissue and phagocytes. Clin Chim Acta. 2019;496:35–44. 31229566 10.1016/j.cca.2019.06.019

[9] Hayatdavoudi P Hosseini M Hajali V Hosseini A Rajabian A. The role of astrocytes in epileptic disorders. Physiol Rep. 2022;10:e15239. 35343625 10.14814/phy2.15239 PMC8958496

[10] Kucukarslan S Reeves AL McAuley JW. Patient-perceived risk associated with epilepsy and its medication treatment. Epilepsy Behav. 2008;13:449–53. 18617018 10.1016/j.yebeh.2008.06.012

[11] Chen M Wu X Zhang B Shen S He L Zhou D. Associations of overweight and obesity with drug-resistant epilepsy. Seizure. 2021;92:94–9. 34481323 10.1016/j.seizure.2021.07.019

[12] Nazish S. Obesity and metabolic syndrome in patients with epilepsy, their relation with epilepsy control. Ann Afr Med. 2023;22:136–44. 37026193 10.4103/aam.aam_139_22 PMC10262857

[13] Kovács R Gerevich Z Friedman A. Bioenergetic mechanisms of seizure control. Front Cell Neurosci. 2018;12:335. 30349461 10.3389/fncel.2018.00335 PMC6187982

[14] Müller MJ Enderle J Bosy-Westphal A. Changes in energy expenditure with weight gain and weight loss in humans. Curr Obes Rep. 2016;5:413–23. 27739007 10.1007/s13679-016-0237-4 PMC5097076

[15] Cressie N. Statistics for Spatial Data. John Wiley & Sons; 2015.

[16] Söylemez E Öztürk O Baslo SA Balçik ZE Atakli D. Metabolic syndrome and obstructive sleep apnea syndrome among patients with epilepsy on monotherapy. Epilepsy Behav. 2020;111:107296. 32769040 10.1016/j.yebeh.2020.107296

[17] Ndayambaje FX Gahutu JB Rugera SP Natukunda B. Prevalence and risk factors for the metabolic syndrome among patients with epilepsy attending a neuropsychiatric hospital in Kigali, Rwanda. Int J Adv Sci Res Manag. 2021;6:1–8.

[18] Beyene Kassaw A Tezera Endale H Hunie Tesfa K Derbew Molla M. Metabolic syndrome and its associated factors among epileptic patients at Dessie Comprehensive Specialized Hospital, Northeast Ethiopia; a hospital-based comparative cross-sectional study. PLoS One. 2022;17:e0279580. 36580471 10.1371/journal.pone.0279580 PMC9799290

[19] Khuda IE Nazish S Zeeshan MA Shariff E Aljaafari D Alabdali M. Non-HDL cholesterol, obesity, and metabolic syndrome in epileptic patients. Prim Care Companion CNS Disord. 2022;24:41350. 10.4088/PCC.21m03057 35687885

[20] Vooturi S Jayalakshmi S. Metabolic syndrome in people with epilepsy. Epilepsy Behav. 2020;106:106992. 32169598 10.1016/j.yebeh.2020.106992

[21] Kegler A Cardoso AS Caprara ALF. Involvement of MnSOD Ala16Val polymorphism in epilepsy: a relationship with seizure type, inflammation, and metabolic syndrome. Gene. 2019;711:143924. 31212050 10.1016/j.gene.2019.06.014

[22] Shan Y Chen Y Gu H Wang Y Sun Y. Regulatory basis of adipokines leptin and adiponectin in epilepsy: from signaling pathways to glucose metabolism. Neurochem Res. 2023;48:2017–28. 36797447 10.1007/s11064-023-03891-2 PMC10181973

[23] Xu L Zhao Z Zhang Y Ma C. The interplay between metabolism and neuroinflammation in epilepsy: mechanisms and therapeutic perspectives. J Neuroinflammation. 2025;22:265. 41220033 10.1186/s12974-025-03553-w PMC12604179

[24] Li YX Chen RX Lv XR Zi Q Zhang FM Li Y. Investigation of the relationship between body mass index and epilepsy. Epilepsy Behav. 2025;165:110295. 40020593 10.1016/j.yebeh.2025.110295

[25] Shnayder NA Grechkina VV Trefilova VV. Valproate-induced metabolic syndrome. Biomedicines. 2023;11:1499. 37239168 10.3390/biomedicines11051499 PMC10216246

[26] Genc BO Dogan EA Dogan U Genc E. Anthropometric indexes, insulin resistance, and serum leptin and lipid levels in women with cryptogenic epilepsy receiving topiramate treatment. J Clin Neurosci. 2010;17:1256–9. 20598547 10.1016/j.jocn.2010.01.045

[27] Isojärvi JI Rättyä J Myllylä VV. Valproate, lamotrigine, and insulin‐mediated risks in women with epilepsy. Ann Neurol. 1998;43:446–51. 9546324 10.1002/ana.410430406

[28] Mahmood IH. Effects of carbamazepine on blood pressure, serum glucose concentration, lipid profile and prevalence of metabolic syndrome in epileptic patients. Tikrit J Pharm Sci. 2012;8:248–55.

[29] Yu H Young JG Petimar J. Long‐term medication‐induced weight change across common antiseizure medications: a target trial emulation study. Obesity (Silver Spring). 2025;33:2355–64. 41047554 10.1002/oby.24362

[30] Chochoł P Arturo N Łajczak PM Rizwan Ahmed A Koppanatham A Varkey TC. Effects of antiseizure medications on lipid profile and weight in patients with epilepsy: a systematic review with meta-analysis. CNS Drugs. 2025;39:1221–39. 41032224 10.1007/s40263-025-01231-2 PMC12602656

[31] Verrotti A Scaparrotta A Agostinelli S Di Pillo S Chiarelli F Grosso S. Topiramate-induced weight loss: a review. Epilepsy Res. 2011;95:189–99. 21684121 10.1016/j.eplepsyres.2011.05.014

[32] Merideth CH. A single-center, double-blind, placebo-controlled evaluation of lamotrigine in the treatment of obesity in adults. J Clin Psychiatry. 2006;67:258–62. 16566621 10.4088/jcp.v67n0212

[33] Isojärvi JI Huuskonen UE Pakarinen AJ Vuolteenaho O Myllylä VV. The regulation of serum sodium after replacing carbamazepine with oxcarbazepine. Epilepsia. 2001;42:741–5. 11422328 10.1046/j.1528-1157.2001.34699.x

[34] Sarangi SC Pattnaik SS Dash Y Tripathi M Velpandian T. Is there any concern of insulin resistance and metabolic dysfunctions with antiseizure medications? A prospective comparative study of valproate vs. levetiracetam. Seizure. 2024;121:123–32. 39146708 10.1016/j.seizure.2024.08.003

[35] Li X Zhai Y Zhao J. Impact of metabolic syndrome and it’s components on prognosis in patients with cardiovascular diseases: a meta-analysis. Front Cardiovasc Med. 2021;8:704145. 34336959 10.3389/fcvm.2021.704145 PMC8319572

[36] Du SM Ma GS Li YP. Relationship of body mass index, waist circumference and cardiovascular risk factors in Chinese adult. Biomed Environ Sci. 2010;23:92–101. 20514983 10.1016/S0895-3988(10)60037-2

[37] Kang G Guo L Guo Z. Impact of blood pressure and other components of the metabolic syndrome on the development of cardiovascular disease. Circ J. 2010;74:456–61. 20057161 10.1253/circj.cj-09-0422

[38] Kim H-L Chung J Kim K-J. Lifestyle modification in the management of metabolic syndrome: statement from Korean Society of CardioMetabolic Syndrome (KSCMS). Korean Circ J. 2022;52:93–109. 35128848 10.4070/kcj.2021.0328 PMC8819565

